# Training of vertical versus horizontal reading in patients with hemianopia – a randomized and controlled study

**DOI:** 10.1007/s00417-020-04952-w

**Published:** 2020-11-04

**Authors:** S. Kuester-Gruber, P. Kabisch, A. Cordey, H.-O. Karnath, S. Trauzettel-Klosinski

**Affiliations:** 1 Vision Rehabilitation Research Unit, Center for Ophthalmology, University of Tübingen, Tübingen, Germany; 2 Center of Neurology, Division of Neuropsychology, Hertie-Institute for Clinical Brain Research, University of Tübingen, Tübingen, Germany

**Keywords:** Homonymous hemianopia, Hemianopic field defect, Reading training, Text orientation, Vertical reading, Hemianopic reading disorder

## Abstract

**Hypothesis:**

Patients with hemianopic field defects (HFD) might benefit from reading text in vertical orientation if they place the text in the seeing hemifield along the vertical midline.

**Methods:**

We assigned 21 patients with HFD randomly to either vertical or horizontal reading training. They trained reading single lines of texts from a computer screen at home for 2 × 30 min/day, 5 days/week, for 4 weeks. The main outcome variable was reading speed (RS) during reading standardized paragraphs of printed text (IReST) aloud. RS was assessed before training (T1), directly after training (T2) and 4 weeks later (T3). Quality of life (QoL) was assessed by Impact of Visual Impairment (IVI) questionnaire.

**Results:**

Vertical training improved RS in the vertical direction significantly. Only patients with right HFD benefited. Horizontal training improved RS in horizontal diection significantly, but much more in patients with left than in those with right HFD. Both effects remained stable at T3. RS during training at the computer improved highly significantly and correlated strongly with RS of printed text (Pearson r= > 0.9). QoL: Vertical training showed a statistically significant improvement in the complete IVI-score, patients with right HFD in the emotional IVI-score.

**Conclusions:**

The improvements of RS were specific for the training. The stable effect indicates that the patients can apply the newly learned strategies to everyday life. The side of the HFD plays an essential role: Left-HFD patients benefitted from horizontal training, right-HFD patients from vertical training. However, the vertical RS did not reach the level of horizontal RS.

The study was registered in the German Clinical Trials register (DRKS-ID: DRKS00018843).

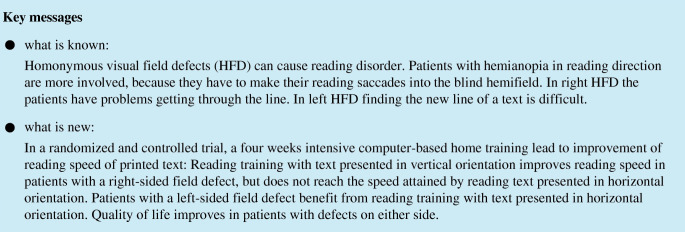



## Introduction

Normal reading requires not only sufficient resolution of the retinal locus used for reading, but also a sufficient size of a reading visual field [1] or visual span [2] during one fixation. This corresponds to the letter recognition span - without eye movements. Reading in the **horizontal direction** requires an asymmetrical strip of visual space, the “perceptual span” that extends 14–15 letters or 5° to the right of fixation and 3–4 letters or 1–2° to the left [3, 4], which is necessary for guiding the next reading saccade to the next letter complex. The concept is based on actual reading data and has been validated by showing that in languages that are read from right to left, this asymmetry is reversed [5–7].

**Fig. 1:**
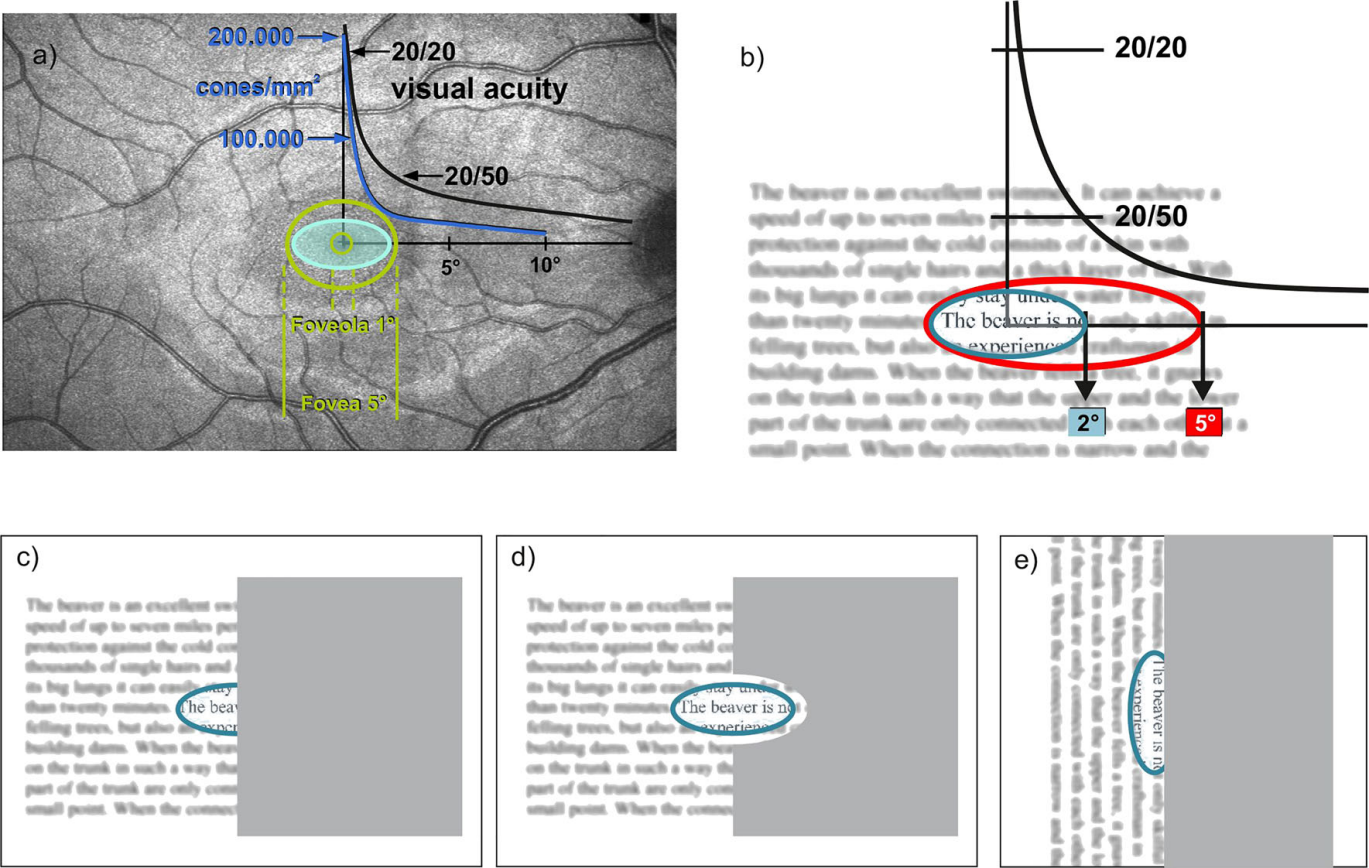
Visual and perceptual span in normal conditions (top) and in patients with hemianopic field defects (HFD). **Top**: **a)** Visual acuity (black) and cone density (blue) depending on eccentricity, the proportions of fovea and foveola (green) and the minimum reading visual field (2° to right and left of fixation and 1° above and below [1]) or visual span or letter recognition span [2]) displayed as turquois oval. **b)** These data related to a text: Due to the decreasing visual acuity curve (black), the letters are seen clearly only within the turquois oval. The perceptual span during a fixation can be increased up to 5° or 15 letters in reading direction by parafoveal information processing [3]. **Bottom**: In patients with HFD, reading ability depends on the distance of the field defect to the center: **c)** in macular splitting, half of the reading visual field is covered by the scotoma and reading is extremely impaired. **d)** In macular sparing, reading can be preserved. **e)** Text rotation and vertical reading might be of functional benefit, as patients can shift the text into the seeing hemifield (modified after [8]).

A homonymous hemianopic field defect (HFD) limits the extent of the perceptual span by partly covering the reading visual field by the scotoma. We have shown in a previous study on patients with hemianopia that a perceptual span of >5° is necessary for fluent reading [8, 9]. In addition, we have also shown that a macular sparing plays an important role for reading ability in HFD [10]. Patients with right HFD have problems progressing through the line, because they have to perform saccades into their scotoma. Patients with left HFD have difficulties finding the beginning of the next line [9]. Hence, it is much more impairing if the HFD lies in the reading direction (Fig. 1).

Reading in **vertical direction** has been studied in healthy subjects and was found to be much slower than in horizontal orientation for languages that are read horizontally, e.g. English, German, Finnish [11–14]. This could be caused by the slightly steeper decline of resolution along the vertical meridian [15], the smaller visual span [1, 16] in the vertical axis, and by a stronger influence of crowding [16, 17]. Further reasons could be the unfamiliar procedure that requires recognizing uncommon word shapes and performing vertical reading saccades.

For patients with age-related macular degeneration with a preferred retinal locus left of the scotoma, training with 90° rotated text presented with RSVP, improved vertical reading speeds, but not more than in horizontal reading [18]. However, for patients with hemianopia, the vertically oriented text might provide a functional benefit, because the patients can place the text in the seeing hemifield along the vertical midline. This was assumed by several authors [8, 10, 14, 19–21] and was examined in a single session (i.e. without training) in hemianopic patients by de Jong et al. [22]. They observed a certain benefit for patients with right HFD, but not for those with left HFD. Hepworth et al. [20] examined 7 hemianopic patients with vertical reading (without training) and found vertical reading speeds slower than in horizontal reading (except one patient with only a partial hemianopia). This raises the question whether systematic training of vertical reading could improve reading performance. The present study is the first randomized and controlled trial (RCT) that applies training of vertical reading in hemianopic patients in order to assess a potential benefit during reading vertically oriented text. We hypothesized that training to read vertically oriented text would improve reading in patients with HFD.

### Study design

**Fig. 2:**
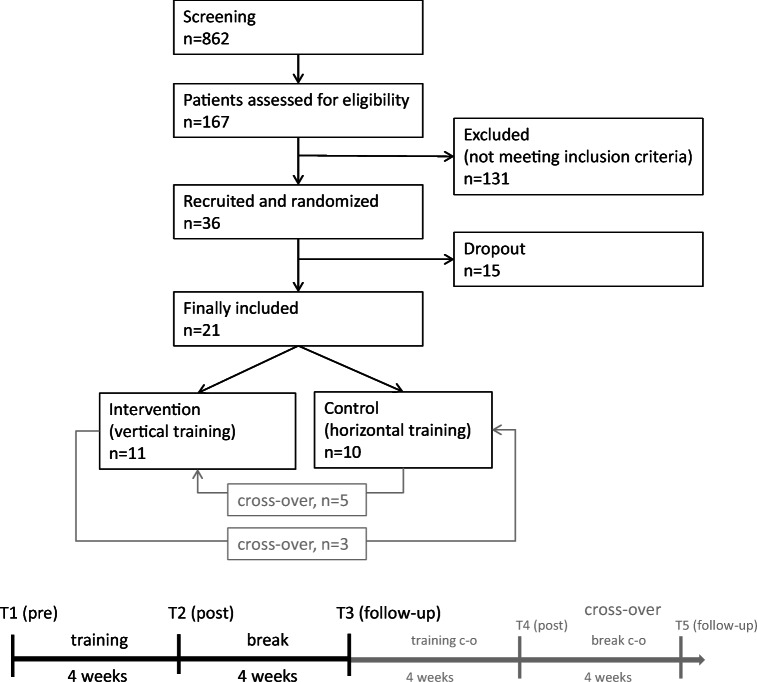
Study design. The testing times were: T1: before training, T2: testing directly after 4 weeks of training, T3: 4 weeks after end of training and beginning of cross-over training

In this RCT, patients were randomly assigned to either of 2 training groups: vertical reading training (group V, *n* = 11) and horizontal reading training (group H, *n* = 10). The latter was used as a control group to address the question of transfer from one reading direction to the other. The horizontal training was initially supposed to be a placebo training. Due to the difficult recruitment situation, we offered a cross-over from the horizontal to the vertical training group and vice versa. However, as only few patients agreed to another training period and two additional visits in the clinic, the cross-over groups became too small, so that we did not include them in the data analysis. The study design is shown in Fig. 2.

## Methods

### Patients

Of the 36 finally recruited patients, 15 dropped out due to the following reasons:

Suspected alexia (2), aphasia (2), not having shown up after training (5), developmental dyslexia (1), insufficient training intensity (3), AMD (1), or personal reasons (1).

Twenty-one HFD- patients, i.e. hemianopia (left, *n* = 11; right *n* = 9) or quadrantanopia (upper right quadrant, *n* = 1) participated in the study (examination period: 07 March, 2016 until 20 August, 2018). All patients had a macular sparing of ≤5 degrees. Macular splitting was found in 10 patients, a sparing of 1° in 7 patients, of 2° in 2 patients, of 4° and 5° in one patient each (for details, see microperimetry, below).

Inclusion criteria:

- HFD for at least 6 months to avoid interference with spontaneous recovery
- macular sparing of equal or less than 5 degrees to include only patients with limited reading visual field
- normal or nearly normal cognitive ability (MoCA scale = > 18)
- visual acuity of at least 0.6 (0.2 LogMAR)
- reading speed <150 words per minute
- German as mother tongue
- ability to consent

Exclusion criteria:

- Additional visual field defects
- any eye disease, except mild cataract
- neglect (assessed by line dissection test)
- no interest in reading
- insufficient co-operation
- aphasia, alexia
- pre-existing reading impairment

These strict inclusion and exclusion criteria made sure that the final group was quite homogeneous, but they also made recruitment very difficult.

This study was conducted in agreement with the tenets of the declaration of Helsinki, and all patients gave their written informed consent for their participation. The project was approved by the ethics committee of the medical faculty of the University of Tübingen, Germany. The study was registered in the German Clinical Trials register (DRKS-ID: DRKS00018843).

## Patient examinations

All patients underwent complete clinical neuro-ophthalmologic and orthoptic examinations, which included tests of best-corrected visual acuity (far/near), binocular status, motility, and eye morphology.

### Specific examinations

Table 1 shows all outcome variables as an overview and the schedule of their assessment. The outcome variables were assessed before training (T1, pre), after the 4 weeks of training (T2, post), and 4 weeks after the end of the training (T3, follow-up). RS was the main outcome variable. The follow-up examination assessed whether the potential training effect remained stable.

**Table 1:** study schedule: examinations in the clinic

**examinations**	**T1**	**T2**	**T3**
**ophthalmological examinations**			
orthoptics	+		
optimal correction	+		
morphology	+		
neuroophthalmological examination	+		
perimetry TwinField	+		(+)
microperimetry	+		
line bisection test	+		
**reading speed measurements**			
**printed text (IReST)**			
text presented vertically (group V)	+	+	+
text presented horizontally (group V and group H)	+	+	+
**quality of life and cognition**			
QoL (IVI)	+	+	+
cognition (MoCA)	+	+	+
questionnaire after the training		+	
reading questionnaire	+	+	+

## Reading speed (RS)

RS was measured in two different settings:

- The main outcome variable was reading speed (RS) in words per minute (wpm) during reading standardized paragraphs of printed text aloud (International Reading Speed Texts, IReST [23, 24], German version) measured in the clinical setting at three time points.

During vertical reading, upcoming text beyond the actually read line was covered by a sheet of paper. The question was, whether a potential effect of the training would transfer to the everyday reading situation. Therefore, this test was performed binocularly. Testing with IReST was performed at all three time points (see Table 1). Patients who had trained reading single lines in vertical orientation, read the IReST paragraphs in vertical and horizontal orientation. Patients who had trained reading single horizontal lines of text read the test paragraphs only in horizontal orientation. For each visit and each task, different IReST paragraphs were used, all of which belonged to the same performance category [23].

b Reading speed in wpm during the home training at the computer (cRS) was calculated and stored by the software (details see below).

In group V, the individually preferred reading orientation of the vertical line was determined during the baseline examination. The two line orientations were from top to bottom with an angle of rotation of 90° to the right (for reading downward), or from bottom to top with an angle of rotation angle of 270° to the right (for reading upward). The duration of a reading session was measured with the software from the start of the reading program until the user pressed the exit button.

## SLO-Microperimetry

The size of macular sparing was examined by a Scanning Laser Ophthalmoscope (SLO, Rodenstock 101, Munich, Germany) in combination with semi-automated custom-designed software. Patients with a sparing of >5° were excluded. The patients were instructed to fixate the central fixation cross (36 arcmin), while the stimuli were presented within five degrees horizontally on the blind side, and 2 degrees horizontally on the seeing side, and 2 degrees vertically in both directions. The stimulus grid was 1 degree. The examiner encouraged the patient continuously to maintain central fixation. If fixation was not central and the answer was ambiguous, the stimulus presentation was repeated. The examination was performed monocularly on both eyes.

## Questionnaires

- Quality of Life (QoL)

We used the German version of the 28-item Impact of Visual Impairment (IVI) questionnaire [25–27]. The possible answers to the questions are Likert-scaled and have the following options: “not at all” (0), “occasionally” (1), “often” (2), “very often” (3), and “I don’t do this because of other reasons” (8). The summary scales of the German version of the IVI-questionnaire differ from the summary scales of the original version. The German version was divided into 2 summary scales: “Functional IVI” and “Emotional IVI”, plus the score for the total questionnaire “Complete IVI” (see [27]).

b) Specific questions regarding reading

We asked the patients specific questions regarding their reading behavior, comfort and amount /duration/intensity at the 3 time points.

c) Spontaneous feedback regarding the training program

After the training period (at T2), we asked the patients about their experience during the training (user-friendliness, difficulties, general acceptance of the program, comfort, additional comments).

d) Cognitive Status

We used the Montreal cognitive assessment (MoCA) [28]) test to judge cognitive ability. It was used primarily as a possible exclusion criterion for patients with major cognitive impairment, but was also applied at post-training and follow-up examinations to assess potential changes. We included patients with either only mild (MoCA-score 18–25) or no cognitive deficit (MoCA-score > 25).

## Training

The training consisted of reading texts that were presented as single lines of text at a time in either horizontal or vertical orientation on the computer screen. The patients could choose different texts from the various categories that were provided together with the training program. Alternatively, they could download any text of interest from the internet. The training software transformed the text into single lines in the required orientation.

The patients were instructed to use the training at home for 30 minutes, twice a day, on 5 days a week, for 4 weeks. Patients who did not own a PC were supplied with a laptop computer by our department. The program started with the request to load a text into the program. Then the text was displayed line by line, centered on the screen, either horizontally or vertically from top to bottom or vice versa. The patient went to the next text line by pressing a button (right arrow key or space bar). The patient could also move backwards line by line with the left arrow key.

The training intensity, i.e. the time the patients spent with the training, was derived from the software as sum of all reading times [in minutes] during the training period.

## Statistical methods

We used IBM SPSS Statistics, version 25, for statistical analyses. If data were normally distributed, parametric confirmatory statistical analyses were applied (paired t-test, t-test for independent samples). In all other cases, we used non-parametric methods. Descriptive data were presented as medians with their interquartile range (IQR). We also used the Wilcoxon signed-rank test, Friedman test, and the Mann-Whitney U test for independent samples. With 3 test points and a significant Friedman test result, we did not perform a Bonferroni correction because it is not indicated (“closed testing procedure” [29]).

The required level of significance α was set to 0.05 (two-sided) in all statistical tests. Unless otherwise stated, given a sample size of 21, the Shapiro-Wilk normality test and graphical Q-Q plots were used to determine the shapes of distributions. All calculations were performed with listwise exclusion (see https://libguides.library.kent.edu/SPSS/Explore).

 Mean imputation was performed in the IVI questionnaire for missing values, as IVI scores are summed up values, and not less than 89% of the scale values were available for each patient [30]*.* For correlations, we calculated the Pearson correlation coefficient r. For the cRS, the reading time at the beginning of the training, the median reading time of all read lines of the first three days of the training period was extracted. The reading time at the end of training was determined to be the median reading time of all read lines of the last 3 days of the training period. By extracting the median, extreme reading times from the training data could be removed. Extreme reading times were possible in two situations: If the patient pressed the forward key to scroll through the text without reading the text and if the patient paused during the reading training without interrupting the program.

The data were analyzed by training group (V vs. H) and side of the HFD (left vs. right).

Median individual changes (delta RS between T1 and T2) of the subgroups were calculated and the individual changes of each patient are presented as scatter-plots.

The statistical values are shown in Table 2.

**Table 2:** **Statistical data.** RS = reading speed of printed text (IReST), cRS = Reading speed during home training at the computer; V = vertical training group, H = horizontal training group; wpm = words per minute**;** HFD = homonymous field defect; IQR = interquartile range; ft = Friedman test; w = post hoc Wilcoxon signed-rank test; tt = pairwise t-test; t() = t-test statistics; CI = confidence interval; M = difference of means; d = Cohen’s measure of effect size; p = significance value; **The impact of vision impairment questionnaire** (**IVI)**: The answers are Likert-scaled and have the following options: “not at all” (0), “occasionally” (1), “often” (2), “very often” (3), and “I don’t do this because of other reasons” (8). The IVI contains 3 subscales: functional, emotional and complete. **MoCA:** Mild cognitive impairment (18–25), no cognitive deficit (>25)

	t1	t2	t3	
	median (IQR) - listwise exclusion			Test - listwise exclusion
**reading speed horizontal [wpm]**				
RS (horizontal) (group V, *n* = 11)	99.00 (63.00–128.00)	99.28 (77.00–143.00)	115.00 (73.60–131.40)	tt, *p* > 0.05
RS (horizontal) (group H, *n* = 10)	112.50 (67.38–123.00)	127.40 (104.00–144.78)	124.50 (71.50–144.56)	ft(t1-t2-t3): χ2(2) = 9.800, ***p*** **= 0.007** w(t1-t2): Z = −1.10, ***p*** **= 0.014** w(t1-t3): Z = −1.30, ***p*** **= 0.004**
RS (horizontal) (HFD left, *n* = 11)	113.00 (80.00–120.00)	129.80 (99.28–144.00)	126.00 (82.50–141.00)	tt(t1-t2), M = −18.40, 95% CI [−30.46, −6.33], t(10) = −3.40, ***p*** **= 0.07**, d = 1.02
RS (horizontal) (HFD right, *n* = 10)	104.50 (47.78–129.00)	109.75 (57.23–143.20)	119.00 (59.00–130.35)	tt(t1-t3), M = −7.30, 95% CI [−13.15, −1.44], t(9) = −2.82, ***p*** **= 0.020**, d = 0.89
cRS (horizontal) (group H, *n* = 10)	126.72 (74.69–142.69)	148.70 (82.69–165.79)	–	tt(t1-t2), M = −18.91, 95% CI [−38.07, 0.23], t(9) = −2.23, *p* = 0.052, d = 0.71
**reading speed vertical [wpm]**				
RS (vertical) (group V, *n* = 11)	90 (44.56–112.00)	104 (56.50–116.40)	104 (58.20–111.00)	tt(t1-t2), M = −9.64, 95% CI [−15.91, −3.36], t(10) = −3.42, ***p*** **= 0.007**, d = 1.03 tt(t1-t3), M = −9.60, 95% CI [−15.69, −3.51], t(10) = −3.51, ***p*** **= 0.006**, d = 1.06
RS (vertical) (HFD left, *n* = 5)	70 (43.03–116.00)	77 (57.51–130.70)	73 (58.80–121.20)	tt, *p* > 0.05
RS (vertical) (HFD right, *n* = 6)	93.85 (46.66–102.04)	104.50 (47.44–110.25)	104.70 (49.21–116.25)	tt(t1-t2), M = −6.77, 95% CI [−12.82, −0.72], t(5) = −2.88, ***p*** **= 0.035**, d = 1.17 tt(t1-t3), M = −10.11, 95% CI [−18.76, −1.47], t(5) = −3.01, ***p*** **= 0.030**, d = 1.23
cRS (vertical) (group V, *n* = 11)	72.93 (39.19–103.55)	103.35 (48.30–118.20)	–	tt(t1-t2), M = −16.31, 95% CI [−24.81, −7.82], t(10) = −4.23, ***p*** **= 0.002**, d = 1.29
**quality of life (IVI)**				
Functional IVI (all patients, *n* = 21)	1.45 (0.55–1.88)	1.05 (0.48–1.84)	1.05 (0.58–1.85)	tt(t1-t2), M = −0.19, 95% CI [0.02, 0.37], t(20) = 2.29, ***p*** **= 0.033**, d = 0.50 tt(t1-t3), M = 0.14, 95% CI [−0.01, 0.29], t(20) = 1.90, *p* = 0.071, d = 0.42
Reading IVI (all patients, *n* = 21)	1.5 (1.00–2.00)	1.0 (0.50–1.50)	1.00 (0.50–1.50)	ft(t1-t2-t3): χ2(2) = 9.042, ***p*** **= 0.011** w(t1-t2): Z = 0.600, *p* = 0.054 w(t1-t3): Z = 0.62, ***p*** **= 0.045**
Reading IVI (HFD right, *n* = 10)	1.75 (0.75–2.00)	1.0 (0.50–1.50)	1.25 (0.38–1.63)	ft(t1-t2-t3): χ2(2) = 5.30, p = 0.07
Emotional IVI (HFD right, *n* = 10)	0.81 (0.39–1.59)	0.86 (0.22–1.11)	0.75 (0.22–1,05)	tt(t1-t3), M = 0.26, 95% CI [0.05, 0.46], t(9) = 2.81, **p = 0.02**, d = 0.89
Complete IVI (all patients, *n* = 21)	1.25(0.57–1.73)	1.00 (0.48–1.63)	1.11 (0.55–1.70)	tt(t1-t2), M = 0.19, 95% CI [0.04, 0.35], t(20) = 2.62, ***p*** **= 0.016**, d = 0.57 tt(t1-t3), M = 0.14, 95% CI [0.01, 0.28], t(20) = 2.25, ***p*** **= 0.036**, d = 0.49
Complete IVI (group V, *n* = 11)	1.25, (0.93–2.13)	1.00 (0.89–1.61)	1.19 (0.86–1.73)	tt(t1-t2), M = 0.22, 95% CI [0.00, 0.44], t(10) = 2.24, ***p*** **= 0.049**, d = 0.67
**cognitive status**				
MoCA score (group V, *n* = 11)	26.0 (21.0–27.0)	26.0 (22.0–27.0)	27.0 (22.0–29.0)	tt; *p* > 0.05
MoCA score (group H, *n* = 10)	23.5 (21.5–26.0)	23.5 (21.0–23.5)	23.5 (20.0–26.5)	tt; *p* > 0.05
MoCA score (HFD left, *n* = 11)	24.0 (21.0–27.0)	24.0 (22.0–26.0)	24.0 (18.0–28.0)	tt; *p* > 0.05
MoCA score (HFD right, *n* = 10)	25.0 (21.5–26.75)	25.0 (21.0–27.5)	25.5 (22.5–29.0)	tt; *p* > 0.05

## Results

I **Reading speed (RS) during reading printed text (IReST)**

- Reading in vertical orientation

This orientation was tested only in those patients who had trained reading lines in vertical orientation (group V, *n* = 11). The median vertical RS improved by 14 wpm from T1 (90 wpm,) to T2 (104 wpm) and remained stable at T3 (104 wpm) (Fig. 3.1 and Table 2). This difference was statistically significant, however, the *median* of the *individual changes* of RS (delta RS T1 - T2) was only 6.3 wpm (10%). Only 3/11 patients improved by at least 10 wpm (see Fig. 3.3).

**Fig. 3:**
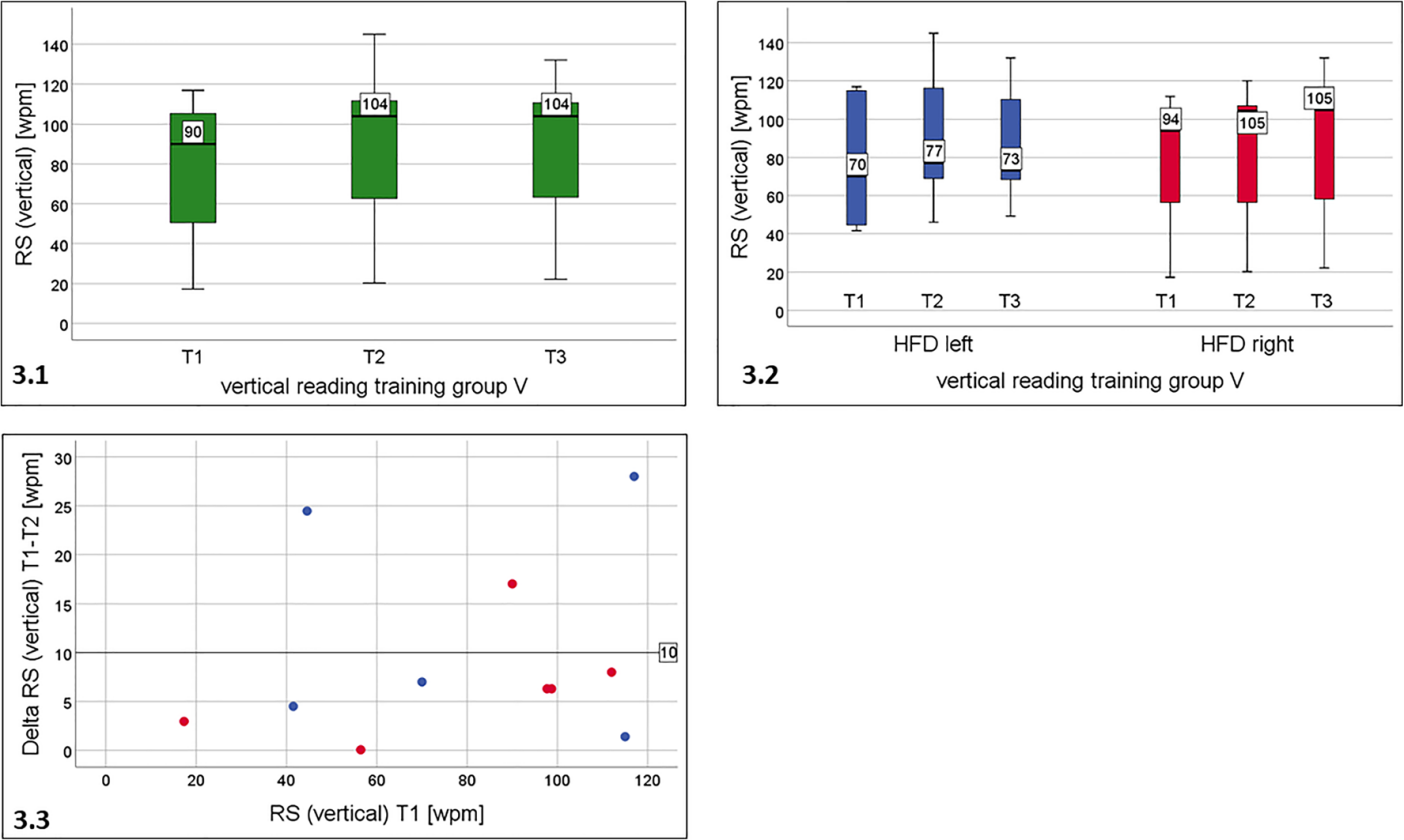
Reading speed for single lines of printed text taken from IReST paragraphs in vertical orientation by patients with vertical reading training (group V). 3.1: For the total group: significant improvement of RS from T1 to T2, which remained stable at T3. 3.2: group V separated into subgroups of left or right hemianopic field defect (HFD): No change in left HFD (*n* = 5), but statistically significant improvement from T1 to T2 in right HFD (*n* = 6). The effect remained stable at T3. 3.3: Individual change of RS (vertical) during the training period: wide overlap between right (red dots) and left HFD (blue dots). Only three patients improved their reading speed by more than 10 wpm (3.3)

Subdividing the groups according to the side of HFD did not yield any clear effects, since the interpretation can be limited by very small subgroups. In patients with left HFD (*n* = 5), there was no significant change: T1: 70 wpm; T2: 77 wpm; T3: 73 wpm. The patients with right HFD (*n* = 6) yielded a statistically significant and clinically relevant improvement by 11 wpm from T1 (94 wpm) to T2 (105 wpm and from T1 to T3 (105, i.e. the training effect remained stable (see Fig. 3.2 and Table 2). Therefore, it appears that only patients with right HFD in group V benefited regarding vertical RS (Fig. 3.2). The *individual change* of RS (vertical) (delta RS T1-T2) shows a wide overlap between right (red dots) and left HFD (blue dots). Only 3 patients (1 right HFD, 2 left HFD) improved by more than 10 wpm (Fig. 3.3). Four patients improved more than 15%, one of them by 55%.

b. Reading text in horizontal orientation

**Total cohort (**Fig. 4.1).

**Fig. 4:**
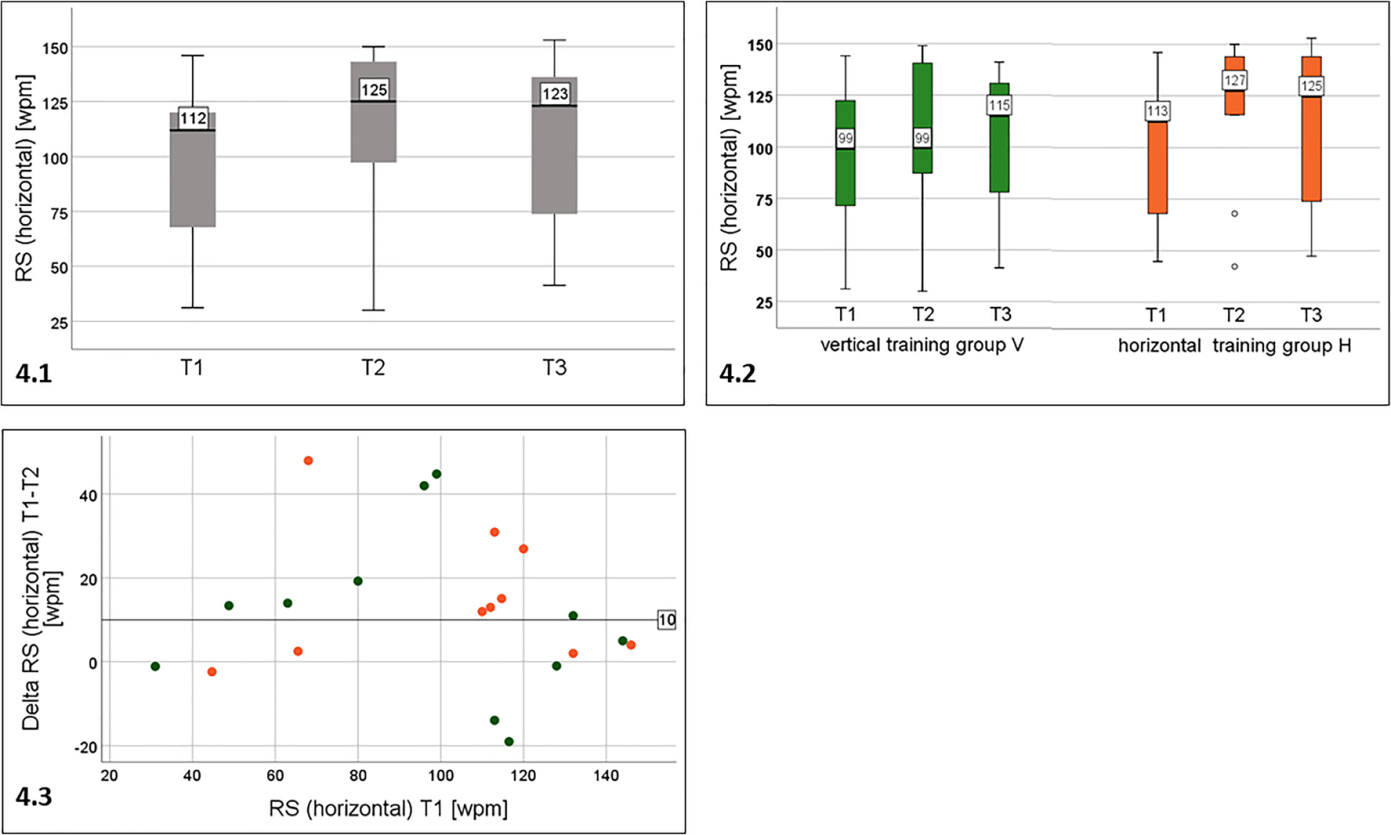
Reading speed for single lines of printed text taken from IReST paragraphs in horizontal orientation. 4.1: all patients (*n* = 21): significant improvement of RS from T1 to T2, which remained stable at T3. 4.2: separated by training groups: no change in the vertical training group V, but statistically significant improvement in the horizontal training group H from T1 to T2 and from T1 to T3. The effect remained stable after training. 4.3: Individual change of RS (horizontal): wide overlap between the groups (group V: green dots, group H: orange dots) without a clear advantage for the horizontal training group

For the total cohort (*n* = 21), there was statistically significant improvement of RS (horizontal) from T1 to T2, which remained stable at T3.

**Separated by training group (**Fig. 4.2–4.3).

Training group V (*n* = 11) did not show any statistically significant changes during horizontal reading.

Training group H (*n* = 10) showed a statistically significant increase of RS of 14.9 wpm from T1 (112.50 wpm) to T2 (127.40 wpm), of 12 wpm from T1 to T3 (124.50 wpm), and no change from T2 to T3, i.e. the training effect remained stable.

The *median* of the *individual changes* of RS (horizontal) (delta RS T1-T2) was 11 wpm for group V and 12.5 wpm for group H.

However, the *individual change* of RS during the training period showed a wide overlap between the groups without a clear advantage for the horizontal training group (see Fig. 4.3). Note that one left-HFD patient of the horizontal training group H improved by 48 wpm (70.6%).

**Fig. 5:**
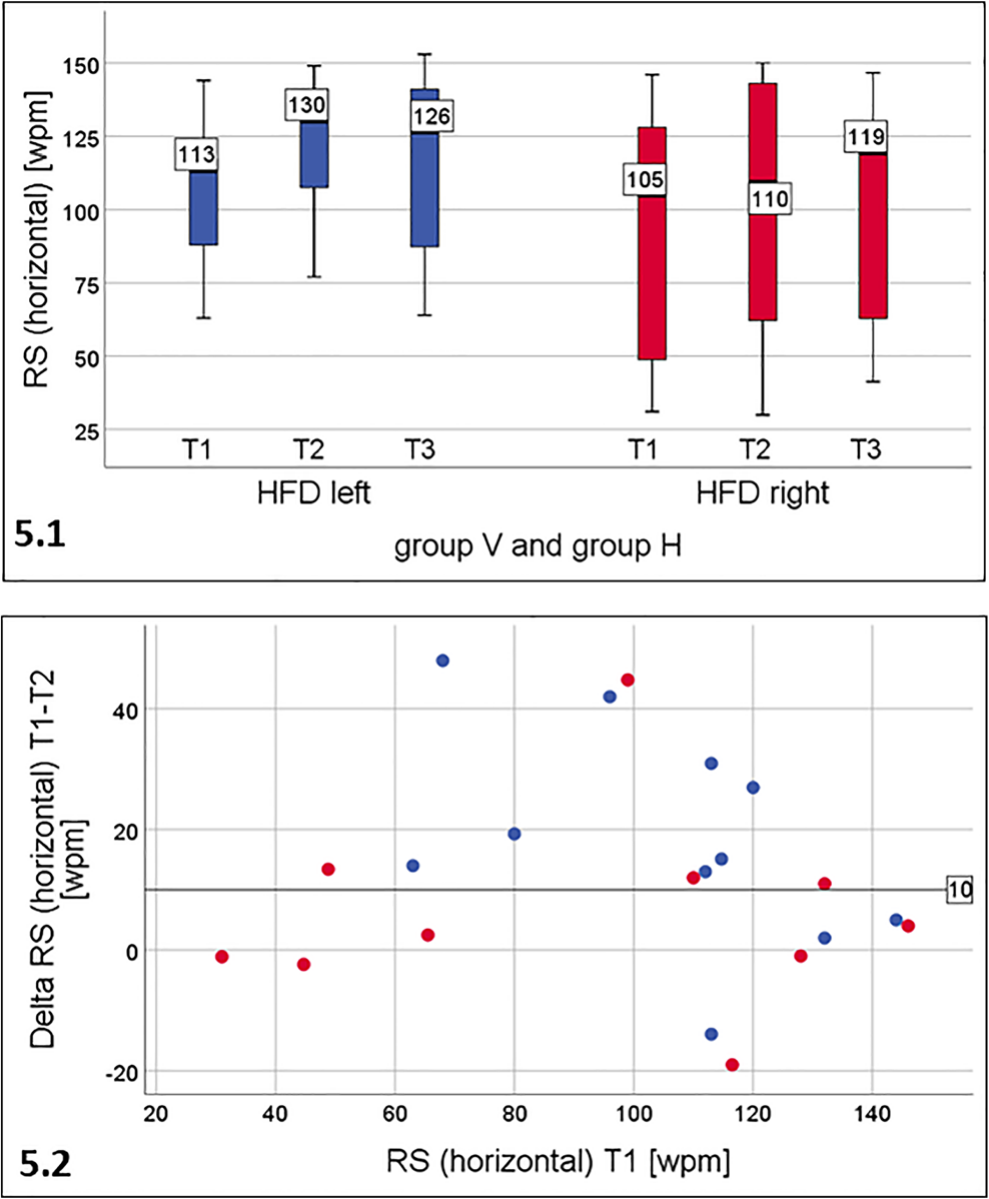
Reading speed for single lines of printed text (IReST) in horizontal orientation. 5.1: total cohort (*n* = 21) separated by the side of the HFD. There was statistically significant improvement in left HFD (*n* = 11) from T1 to T2 (*p* = 0.007), which remained stable. In right HFD (*n* = 10) there was statistically significant improvement only from T1 to T3 (*p* = 0.02), and less pronounced than in left HFD. 5.2: Individual improvement of RS (horizontal) during the training period: patients with left HFD (blue dots) also improved more than those with right HFD (red dots). Eight patients with left HFD showed an improvement of more than 10 wpm, only four patients with right HFD

**Patients separated by the side of HFD (**Fig. 5**):**

Separating all patients (*n* = 21) into groups according to the side of the HFD (independent of the training group), patients with left HFD (*n* = 11) showed a statistically significant increase of median RS during horizontal reading from T1 (113.0 wpm) to T2 (129.8 wpm), see Fig. 5.1. In right HFD the increase of median RS was significant only from T1 to T3.

The *median* of the *individual changes* of RS (delta RS T1-T2) was 15.1 wpm (22.2%) in left HFD and 5.6 wpm [8.8%] in right HFD (delta RS T1-T3).

For the *individual change* (Fig. 5.2), patients with left HFD also showed more improvement than for right HFD. Eight patients with left HFD showed an improvement of more than 10 wpm, only four patients with right HFD. Six left HFD patients showed an improvement of more than 15% of their RS, but only 2 with right HFD.

RS correlated highly with cRS (RS during the training) (see below). No correlations were found between RS at T1 and the change of RS (from T1 to T2), regarding age, disease duration, and size of macular sparing.

II. **Reading speed during the training at the computer (cRS)**

Group V (*n* = 11) showed a statistically significant improvement of the reading speed at the computer during the training (cRS) between the **median** of the first 3 days (72.93 wpm) and the **median** of the last 3 days (103.35 wpm) of the vertical reading training (see Table 2).

Group H (*n* = 10) improved cRS during the horizontal reading training, but this change was not statistically significant: the mean of the first 3 days of training was 126.72 wpm; the mean of the last 3 days of training was 148.70 wpm. (see Table 2).

**Fig. 6:**
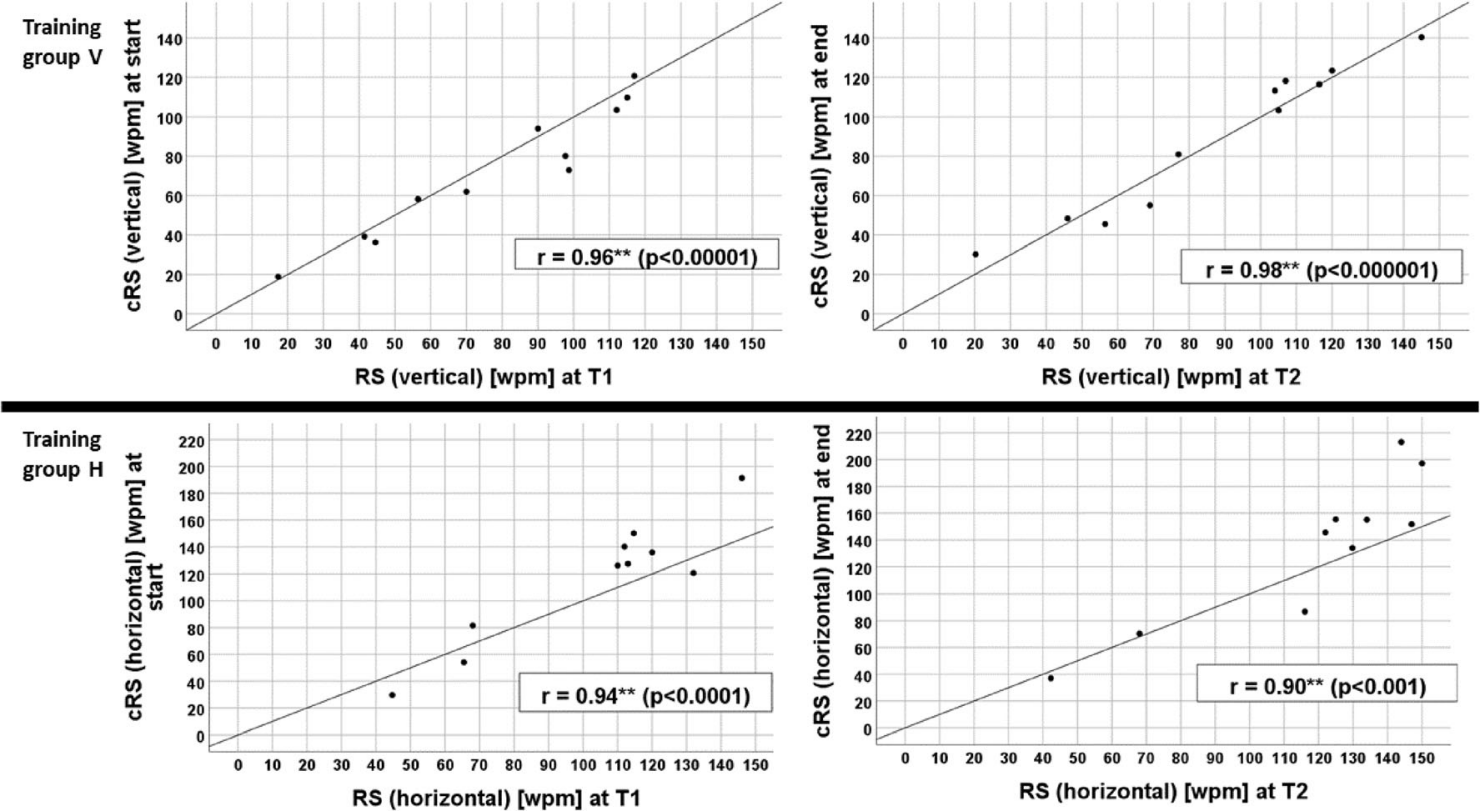
Reading speed during home training at the computer (cRS) vs. reading speed (RS) during testing with printed text (IReST paragraph) in the clinic. Top: group V, vertical reading training at T1 (left) and T2 (right). Bottom: Group H, horizontal reading training at T1 (left) and T2 (right). cRS and RS in both training groups were strongly correlated (see r-values in graph)

cRS (during the home training) correlated highly with RS (at testing in the clinic with the printed IReST charts) (see Fig. 6).

III. **Training intensity**

The study design requested that each patient should train for 4 weeks, 5 days per week, 2 × 30 min per day (1200 min = 20 h). The actually measured median cumulative training time of all patients (*n* = 21) was 1236.45 min (IQR 872.2–1417.4, range 715. 48–1963.1 min) (103%). As the actual average training time per day for each patient was 62 min, this means that there was good compliance. There was neither a statistically significant correlation between training intensity and change of RS (T1-T2), nor between training intensity and change of cRS during the training at home (*n* = 21, all patients).

IV. **Questionnaires**

- **Quality of Life (IVI)**

**Total cohort.**

When all patients were analyzed together (*n* = 21), we found a statistically significant improvement in the functional IVI-score from T1 (1.45) to T2 (1.05), which remained stable at T3 (1.05). The IVI reading-score showed an improvement from T1 (1.5) to T2 (1.0) and from T1 to T3 (1.00), which was statistically marginally significant. The complete IVI-score showed statistically significant improvement from T1 (1.25) to T2 (1.00) and from T1 to T3 (1.11) indicating that the effect remained stable after training. An improvement of the complete IVI-score was found in 11/12 patients who improved their RS by more than 10 wpm, but was found by only 3 patients who had not improved their RS. This indicates that a successful training was considered beneficial in everyday life by the patients.

**When the total cohort was** **separated by** **reading training group**, group V showed a statistically significant improvement in the complete IVI-score from T1 (1.25) to T2 (1.00), group H did not show any significant improvement.

**When the total cohort was separated by side of HFD,** in left HFD, no significant improvement was observed. In right HFD, the improvement was significant in the emotional IVI-score from T1 (0.81, IQR 0.39 to 1.59) to T3 (0.75, IQR 0.22 to 1,05) (*p* = 0.02, t-test). The reading IVI score tended to improve: T1: 1.50 (IQR 1.00 to 2.00); T2: 1.0 (IQR 0.50 to 1.50); T3: 1.00 (IQR 0.50 to 1.00), *p* = 0.07, Friedman’s test. Post-hoc tests were not applied, as Friedman’s test did not show a significant improvement.

b) **Specific questions regarding reading performance**

Some patients reported spontaneously that reading was „fun again“, that they were reading “much more than before”, and that they were reading “more comfortably”. The questionnaire showed that the amount of reading in daily life increased in 6 patients, the strain decreased in 5 patients. This was assessed at T1, T2 and T3.

c) **Feedback regarding the training program**

As not all patients answered all the questions, we can only report the absolute numbers. The questions were asked after the training at T2.

How demanding was the training for the patients: Five patients described the training as slightly demanding (3 for the vertical training, 2 for the horizontal training), 7 patients reported the training to be demanding (4 vertical, 3 horizontal), 4 found it exhausting (3 vertical, 1 horizontal). This effort decreased during the training period in 9 (v3, h6) and remained equal in 10 (v8, h2) patients.

Question regarding the handling of the program: It was easy for 9 and needed help in another 5 patients. Four patients reported difficulties. Reading in daily life was experienced as easier in 5, equal in 13 patients and harder in none.

d) **Cognitive Status**

The MoCA score was not statistically different, neither between the reading training groups nor between the groups divided by the side of the HFD, and did not change during the training period (see Table 2).

## Discussion

The present RCT is the first study that performed vertical reading training in patients with HFD. Our results show that the patients who trained reading in vertical orientation (group V), experienced a statistically significant improvement of their median vertical RS. This effect remained stable at T3, and they improved their RS not only during the training at the computer, but also during reading the printed IReST. This means that the patients applied their improved reading performance to everyday life. The finding that only patients with right HFD benefited regarding vertical RS is in accordance with the finding by de Jong et al. [22]. This is plausible, because patients with right HFD have the problem of having to perform their reading saccades into the blind hemifield while reading horizontally.

Horizontal training (group H) led to statistically significant improvement of RS in horizontal orientation. The effect remained stable at T3. Despite the improvement during vertical training, the vertical RS at T2 (104 wpm) did not reach the level of horizontal RS (T1: 112.5; T2: 127 wpm). It should be taken into account that most people have practiced horizontal reading throughout their lives, whereas vertical reading is unfamiliar and challenging, e.g. because of the perceptually unusual word shapes and the necessity to perform reading saccades in a vertical direction. In an unpublished pilot study, normally sighted subjects found that vertical reading was quite demanding and tiring. However, it should be kept in mind that normally sighted people will not have any functional advantage using a vertically orientated text, whereas HFD-patients might have this advantage by shifting the line of text into their seeing hemifield. Our results allow hypothesizing that these patients might benefit only after much longer training of reading in the vertical direction.

In fact, the difference between horizontal and vertical reading performance disappears, if readers are used to read in both directions, as in Chinese and Japanese [31]. Furthermore, it was reported that reading direction primed Japanese readers to activate the corresponding direction of visual information processing [32]. One could argue that these languages are logographic and the words can be written in both directions without changing their shape (as they keep the same orientation). In a study using event–related potentials, the recognition of Chinese characters was reported to be delayed at occipital–temporal sites, if the orientation of the characters was changed [33]. However, there is an example of an alphabetic script that can be written in horizontal and vertical direction, namely Mongolian, in which the reading direction is always consistent with the orthographic orientation. It was recently reported that the effects of the perceptual span on reading were similar in both directions, which shows the flexibility of the perceptual span [34].

In our total cohort of patients (V + H), we found a statistically significant increase of RS from T1 to T2 for horizontal reading. This effect remained stable at T3. Regarding the side of the HFD, patients with left HFD showed stronger improvements from T1 to T2 than those with right HFD from T1 to T3 during horizontal reading, but not in vertical reading. Patients with right HFD have the problem of having to perform saccades into the blind hemifield, but can at least improve by reading more frequently. The patients with left HFD have the advantage of getting through the line rather easily during horizontal reading and might need some more (unspecific) training of the return sweep to the next line.

Even though we found statistically significant effects in the groups and subgroups, we should consider that the changes of RS were individually very different and did not show very clear effects. One limitation of the current study is the small sample size, which makes it difficult to form subgroups. The recruitment of the patients was very difficult, which is shown in Fig. 2. However, the strict inclusion and exclusion criteria lead to a homogenous patient sample, which can partly compensate for the small patient group. In this study, it was of special importance to include only patients with macular sparing of <=5 degrees. This precondition would also be important for further studies with larger patient groups.

We consider a change of 10 wpm in these patients as clinically relevant. The value is based on our long-standing clinical experience and has been used in previous studies (e.g. [23, 35]). In a recent Cochrane review by Virgili et al. (2013), reading speed differences were reported as improvements in several studies starting with a value of 12 wpm. The value of 10 wpm is also supported by the observation in this study that 11 of 12 patients reported an increase of their quality of life (IVI complete score), who improved their reading speed by more than 10 wpm. However, the relevance of an improvement should be seen in relation to the actual RS. An improvement by 10 wpm is considerable if it occurs in the speed range around 40 wpm, but not if it occurs at reading speeds of 100 wpm and above.

There are two RCTs on reading training in patients with HFD that used different approaches. Spitzyna et al., (2006) [36] applied scrolled text in patients with right HFD and reported an increase of RS, which is to be expected in these patients. Aimola et al. (2014) [37] used a search task in a line of words and found an improvement of RS in left HFD more than in right HFD.

The strong correlation between reading speed during home training at the computer (cRS) with reading speed of printed paragraphs (RS) in the clinic shows that the method of measuring reading speed by software allows an assessment of the training results at home. The advantage is that the training effect can be determined by software without an additional visit in the clinic. This could save additional effort for the patient and lowers the costs for the health insurance, which would allow monitoring the status of patients with reduced mobility by “telemedicine”. The strong correlation between RS during reading printed text and cRS during the home training at the computer shows that the measurement of reading speed in both conditions is reliable and shows that the data is robust. This also indicates that the patients actually read the texts during the training at home without a supervising person.

Not all of the objective improvements were reflected in the subjective reports by the patients. The fact that QoL improved in group V and in right HFD is in accordance with the finding that patients with right HFD and vertical training benefited most.

The cognitive status (MoCA) of our patients did not change. There was neither an increase (by more intensive reading) nor a decrease (by potential deterioration of their general health condition) of the cognitive status of our patients.

In summary, we found significant improvements of reading speed in two groups using reading training in horizontal or vertical orientation. Training in vertical text orientation improved RS in patients with a right hemianopic field defect, but does not reach the speed attained by reading text presented in horizontal orientation.

In patients with a left HFD, the vertical training did not offer an advantage, because their main problem is finding the beginning of the new line, which they can train more easily by horizontal reading.
